# Temporal trends in pre-ART patient characteristics and outcomes before the test and treat era in Central Kenya

**DOI:** 10.1186/s12879-021-06706-3

**Published:** 2021-09-26

**Authors:** P. Wekesa, A. McLigeyo, K. Owuor, J. Mwangi, L. Isavwa, A. Katana

**Affiliations:** 1 Centre for Health Solutions – Kenya, Nairobi, Kenya; 2 Division of Global HIV & TB, Centers for Disease Control and Prevention (CDC), Nairobi, Kenya

**Keywords:** HIV, Pre ART, Characteristics, Treatment, Outcomes, Trends

## Abstract

**Background:**

Retention of patients who did not initiate antiretroviral therapy (ART) has been persistently low compared to those who initiated ART. Understanding the temporal trends in clinical outcomes prior to ART initiation may inform interventions targeting patients who do not initiate ART immediately after diagnosis.

**Methods:**

A retrospective cohort analysis of known HIV-infected patients who did not initiate ART from healthcare facilities in Central Kenya was done to investigate temporal trends in characteristics, retention, and mortality outcomes. The data were sourced from the Comprehensive Care Clinic Patient Application Database (CPAD) and IQ care electronic patient-level databases for those enrolled between 2004 and 2014.

**Results:**

A total of 13,779 HIV-infected patients were assessed, of whom 30.7% were men.There were statisitically significant differences in temporal trends relating to marital status, WHO clinical stage, and tuberculosis (TB) status from 2004 to 2014. The proportion of widowed patients decreased from 9.1 to 6.0%. By WHO clinical stage at enrollment in program, those in WHO stage I increased over time from 8.7 to 43.1%, while those in WHO stage III and IV reduced from 28.5 to 10.8% and 4.0 to 1.1% respectively. Those on TB treatment during their last known visit reduced from 8.3 to 3.9% while those with no TB signs increased from 58.5 to 86.8%. Trends in 6 and 12 month retention in the program, loss to follow-up (LTFU) and mortality were statistically significant. At 6 months, program retention ranged between 36.0% in 2004 to a high of 54.1% in 2013. LTFU at 6 months remained around 50.0% for most of the cohorts, while mortality at 6 months was 7.5% in 2004 but reduced to 3.8% in 2014. At 12 months, LTFU was above 50.0% across all the cohorts while mortality rate reached 3.9% in 2014.

**Conclusion:**

Trends in pre ART enrollment suggested higher enrollment among patients who were women and at earlier WHO clinical stages. Retention and mortality outcomes at 6 and 12 months generally improved over the 11 year follow-up period, though dipped as enrollment in asymptomatic disease stage increased.

## Background

In 2018, there were 36.9 million people living with HIV (PLHIV) globally, and 1.8 million new HIV infections [1]. In the same year, Kenya had 1.49 million PLHIV, with an estimated 1.12 million persons on antiretroviral treatment (ART), translating to a treatment coverage of 75.0% [2] and an unmet ART need in the country of approximately 370,000 PLHIV. This large population of patients not on ART were either undiagnosed or had been diagnosed, but not yet initiated on ART.

Trends in HIV programs suggest higher enrollment numbers among women, the less symptomatic, and a reduction in enrollment of children over time [1, 4]. The Kenya AIDS Indicator Survey of 2012 (KAIS) reported a higher HIV prevalence among women (6.9%) compared to men (4.4%) [3]. The Joint United Nations Programme on HIV/AIDS (UNAIDS) reported a gender gap in new HIV infections in sub-Saharan Africa, with 59.0% of new infections among women [1]. New HIV infections among children below 14 years of age reduced by 38.0% between 2011 and 2017 [4], likely due to the effectiveness of prevention of mother to child transmission (PMTCT) programs. Further, a study in Tanzania reported a progressive reduction in WHO clinical stage at enrollment in more recent years [5].

The global community has embraced the strategy to treat all PLHIV regardless of clinical stage or CD4 count, also known as Universal Test and Treat (UTT) [6]. Since its endorsement by the World Health Organization (WHO) in 2015, several countries adopted the strategy and began implementation in clinical and programmatic settings [7, 8]. Despite the progress, resource constraints may limit implementation in some countries and there is evidence that not all diagnosed PLHIV immediately initiated ART with a 17.0% gap in 2018 [9, 10]. This suggests a need to understand patient and program level gaps in the pre-ART period—time between diagnosis and initiation of ART—to highlight factors that limited successful initiation of ART that may persist in the post UTT era [10].

Patients not on ART have lower retention in HIV care, compared to those on ART [6, 7]. A recent publication demonstrated this retention on ART including among 36-month cohorts in Central Kenya [11]. Prior to the UTT era, retaining patients in care during the pre-ART period had benefits such as prevention of morbidity and mortality, regular clinical and laboratory monitoring, timely initiation of ART and reduced HIV transmission [12]. A systematic review of program retention rates prior to ART initiation reported 12 month retention rates ranging from 23.0 to 88.0% [13]. While some may re-engage at other treatment sites, gaps in reporting systems may result in misclassification of program retention outcomes [10, 11]. Disengagement from care is associated with increased costs related to patient tracing interventions, increased transmission of HIV, and increased risk of morbidity and mortality [14].

This analysis aims to describe trends in patient characteristics and clinical outcomes for pre -ART patients, such as 6 and 12 month program retention, loss to follow-up (LTFU), and mortality over an 11 year period in Central Kenya. This analysis focuses on the period prior to UTT, where eligibility criteria for initiation of treatment varied from CD4 counts of 350–500 cells/μl effected in 2014.

## Methods

### Study setting, period, population and sample

Center for Health Solutions—Kenya (CHS) received funding through the US Centers for Disease Prevention and Control (CDC) to support the implementation of the HIV prevention and treatment programs in the former Central Province of Kenya since 2010. The package of care for patients was standardized across 47 health facilities and was provided by a multidisciplinary team (MDT) composed of clinical officers, nurses, lay counselors, peer educators, nutritionists, social workers, pharmaceutical technologists/pharmacists and laboratory technologists. Quality of care assessments and continuous quality improvement (CQI) projects were routinely conducted to optimize service delivery. The model of care for patients not initiated on ART included same day enrollment, comprehensive clinical evaluation with appropriate management of any diseases, tuberculosis screening using a symptom questionnaire administered during clinic visits, three sessions of pre-ART education and counselling and based on assessed readiness, initiation of ART, baseline CD4 count, weekly follow-up visits, psychosocial support group meetings, peer support, and tracing of those LTFU.

This analysis focused on 13,779 patients enrolled but not initiated on ART between 2004 and 2014 with 6 and 12 month follow up data documented in the Comprehensive Care Clinic Patient Application (CPAD) electronic and IQ care medical records systems. The patients had not initiated ART by 12 months but may have gone on to initiate ART later on. Patients documented as having transferred to other facilities were excluded.

### Variables

Patient level variables collected included age, sex, marital status, baseline World Health Organization (WHO) clinical stage, and tuberculosis (TB) status. CD4 variable was excluded due to large percentage of missing values. Outcome variables were LTFU, program retention, and mortality at 6 and 12 months. LTFU was defined as not having attended a clinic for more than 90 days and retained patients were defined as patients enrolled, still alive, and active in the review period. Determination of mortality and transfers out were based on documentation available in the patient charts.

### Data sources

Routine data collected between January 2004 and 2014, were extracted from CPAD and IQ care electronic medical record (EMR) systems. Data entry from the paper-based Kenyan ministry of health (MOH) longitudinal patient level data tool (MOH 257), was updated daily after patients’ clinical visits into the EMR by trained facility data clerks. Data were routinely reviewed for errors and completeness by the data manager and subsequently cleaned by monitoring and evaluation teams after verification by the clinical teams as per standard CHS monitoring and evaluation procedures.

### Data analysis

Demographic and baseline clinical characteristics were described and presented as counts and respective percentages for all the variables. A nonparametric test for trend across ordered groups was done and corresponding p-values reported to assess for temporal trends in outcomes and baseline characteristics across the pre-ART cohort years. Line graphs were used to visually display the trends in the outcomes. Statistical significance was evaluated at the 5.0% level. All the analyses were done in Stata version 15.1 (StataCorp. 2017. *Stata Statistical Software: Release 15*. College Station, TX: StataCorp LLC.).

## Results

### Patient characteristics

**Fig. 1:**
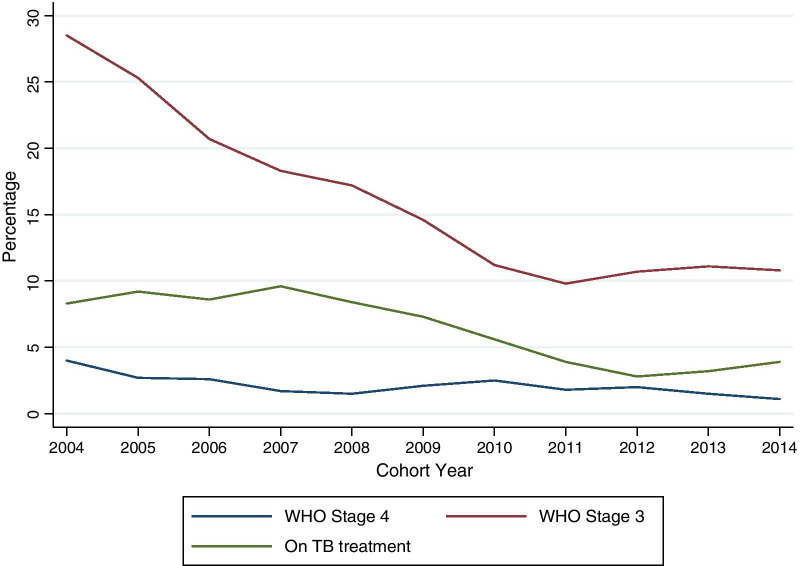
Pre-ART cohort trends from 2004 to 2014

A total of 13,779 pre-ART patients were assessed, out of whom 30.7% were men. Majority of the patients were in age groups 20–35 and 36–50 years across all the cohort years as shown in Table 1. There were statisitically significant differences in patient characteristics over time trends relating to, WHO clinical stage, TB status and, marital status. There was a decrease in the proportion of widowed patients from 9.1% in 2004 to 6.0% in 2014. The proportion of patients enrolled in WHO stage I increased over time from 8.7% in 2004 to 43.1% in 2014 while those enrolled in WHO stage III reduced from 28.5% in 2004 to 10.8% in 2014. Patients enrolled in WHO stage IV also reduced from 4.0% in 2004 to 1.1% in 2014 as shown in Fig. 1. Those on TB treatment during their last known visit reduced from 8.3% in 2004 to 3.9% in 2014 while those with no TB signs increased from 58.5% in 2004 to 86.8% in 2014. Though there was not a statistically significant difference in trend for age as a characteristic, it was notable that the proportion of patients aged between 0 and 4 years reduced from 8.7% in 2004 to 1.1% in 2014 and, similarly, for those aged between 5 and 9 years from 10.7% to 1.0% as shown in Table 1.

**Table 1:** Characteristics of pre-ART cohorts in Central Kenya between 2004 and 2014

	Cohort year											Trend P value
	2004 (n = 254)	2005 (n = 600)	2006 (n = 1019)	2007 (n = 2266)	2008 (n = 2396)	2009 (n = 1992)	2010 (n = 1531)	2011 (n = 1290)	2012 (n = 1000)	2013 (n = 820)	2014 (n = 612)	
*Age*												0.282
0–4 (n = 561)	22 (8.7)	45 (7.5)	73 (7.2)	125 (5.5)	114 (4.8)	59 (3.0)	47 (3.1)	31 (2.4)	21 (2.1)	17 (2.1)	7 (1.1)	
5–9 (n = 552)	27 (10.7)	51 (8.5)	84 (8.2)	138 (6.1)	79 (3.3)	67 (3.4)	43 (2.8)	24 (1.9)	20 (2.0)	13 (1.6)	6 (1.0)	
10–14 (n = 242)	7 (2.8)	15 (2.5)	10 (1.0)	38 (1.7)	29 (1.2)	44 (2.2)	35 (2.3)	18 (1.4)	21 (2.1)	8 (1.0)	17 (2.8)	
15–19 (n = 316)	4 (1.6)	8 (1.3)	12 (1.2)	52 (2.3)	44 (1.8)	34 (1.7)	45 (2.9)	43 (3.3)	26 (2.6)	34 (4.1)	14 (2.3)	
20–35 (n = 7303)	113 (44.7)	263 (43.8)	491 (48.2)	1191 (52.6)	1315 (54.9)	1093 (54.9)	829 (54.1)	698 (54.1)	563 (56.3)	436 (53.2)	311 (50.8)	
36–50 (n = 3803)	58 (22.9)	176 (29.3)	278 (27.3)	572 (25.2)	655 (27.3)	562 (28.2)	437 (28.5)	376 (29.1)	262 (26.2)	229 (27.9)	198 (32.4)	
51 and above (n = 1002)	22 (8.7)	42 (7.0)	71 (7.0)	150 (6.6)	160 (6.7)	133 (6.7)	95 (6.2)	100 (7.8)	87 (8.7)	83 (10.1)	59 (9.6)	
*Sex*												0.398
Female (n = 9551)	160 (63.2)	407 (67.8)	676 (66.3)	1593 (70.3)	1699 (70.9)	1362 (68.4)	1063 (69.4)	891 (69.1)	705 (70.5)	591 (72.1)	404 (66.0)	
Male (n = 4228)	93 (36.8)	193 (32.2)	343 (33.7)	673 (29.7)	697 (29.1)	630 (31.6)	468 (30.6)	399 (30.9)	295 (29.5)	229 (27.9)	208 (34.0)	
*Marital status*												< 0.001
Divorced (n = 1899)	16 (6.3)	49 (8.2)	86 (8.4)	266 (11.7)	291 (12.1)	274 (13.8)	271 (17.7)	243 (18.8)	142 (14.2)	151 (18.4)	110 (18.0)	
Married (n = 5881)	54 (21.3)	156 (26.0)	345 (33.9)	935 (41.3)	1075 (44.9)	914 (45.9)	671 (43.8)	601 (46.6)	471 (47.1)	395 (48.2)	264 (43.1)	
Single (n = 3040)	45 (17.8)	122 (20.3)	188 (18.4)	474 (20.9)	552 (23.0)	457 (22.9)	338 (22.1)	251 (19.5)	237 (23.7)	200 (24.4)	176 (28.8)	
Widowed (n = 1029)	23 (9.1)	45 (7.5)	100 (9.8)	207 (9.1)	189 (7.9)	130 (6.5)	112 (7.3)	87 (6.7)	63 (6.3)	36 (4.4)	37 (6.0)	
Not indicated (n = 1930)	115 (45.5)	228 (38.0)	300 (29.4)	384 (16.9)	289 (12.1)	217 (10.9)	139 (9.1)	108 (8.4)	87 (8.7)	38 (4.6)	25 (4.1)	
*WHO stage*												< 0.001
Stage I (n = 4732)	22 (8.7)	89 (14.8)	151 (14.8)	585 (25.8)	797 (33.3)	706 (35.4)	630 (41.1)	566 (43.9)	519 (51.9)	403 (49.1)	264 (43.1)	
Stage II (n = 3251)	44 (17.4)	108 (18.0)	196 (19.2)	561 (24.8)	609 (25.4)	481 (24.1)	351 (22.9)	311 (24.1)	246 (24.6)	193 (23.5)	151 (24.7)	
Stage III (n = 2113)	72 (28.5)	152 (25.3)	211 (20.7)	414 (18.3)	413 (17.2)	290 (14.6)	171 (11.2)	126 (9.8)	107 (10.7)	91 (11.1)	66 (10.8)	
Stage IV (n = 268)	10 (4.0)	16 (2.7)	26 (2.6)	38 (1.7)	35 (1.5)	42 (2.1)	39 (2.5)	23 (1.8)	20 (2.0)	12 (1.5)	7 (1.1)	
Unstaged (n = 3415)	105 (41.5)	235 (39.2)	435 (42.7)	668 (29.5)	542 (22.6)	473 (23.7)	340 (22.2)	264 (20.5)	108 (10.8)	121 (14.8)	124 (20.3)	
*TB status*												< 0.001
No signs (n = 10,760)	148 (58.5)	373 (62.2)	670 (65.8)	1578 (69.6)	1810 (75.5)	1601 (80.4)	1299 (84.8)	1123 (87.1)	893 (89.3)	734 (89.5)	531 (86.8)	
On TB treatment (n = 943)	21 (8.3)	55 (9.2)	88 (8.6)	218 (9.6)	202 (8.4)	146 (7.3)	85 (5.6)	50 (3.9)	28 (2.8)	26 (3.2)	24 (3.9)	
Presumptive TB (n = 595)	12 (4.7)	6 (1.0)	28 (2.7)	95 (4.2)	130 (5.4)	112 (5.6)	71 (4.6)	50 (3.9)	38 (3.8)	26 (3.2)	27 (4.4)	
Not indicated (n = 1481)	72 (28.5)	166 (27.7)	233 (22.9)	375 (16.5)	254 (10.6)	133 (6.7)	76 (5.0)	67 (5.2)	41 (4.1)	34 (4.1)	30 (4.9)	
*CD4 category*												< 0.001
0–250 (n = 778)	21 (8.3)	25 (4.2)	148 (14.5)	136 (6.0)	109 (4.5)	103 (5.2)	80 (5.2)	48 (3.7)	51 (5.1)	34 (4.1)	23 (3.8)	
251–500 (n = 136)	0 (0.0)	2 (0.3)	16 (1.6)	22 (1.0)	24 (1.0)	16 (0.8)	8 (0.5)	15 (1.2)	11 (1.1)	10 (1.2)	12 (2.0)	
Above 500 (n = 43)	2 (0.8)	0 (0.0)	14 (1.4)	9 (0.4)	9 (0.4)	1 (0.1)	5 (0.3)	0 (0.0)	3 (0.3)	0 (0.0)	0 (0.0)	
No CD4 test (n = 12,822)	230 (90.9)	573 (95.5)	841 (82.5)	2099 (92.6)	2254 (94.1)	1872 (94.0)	1438 (93.9)	1227 (95.1)	935 (93.5)	776 (94.6)	577 (94.3)

### Outcomes at 6 and 12 months

**Fig. 2:**
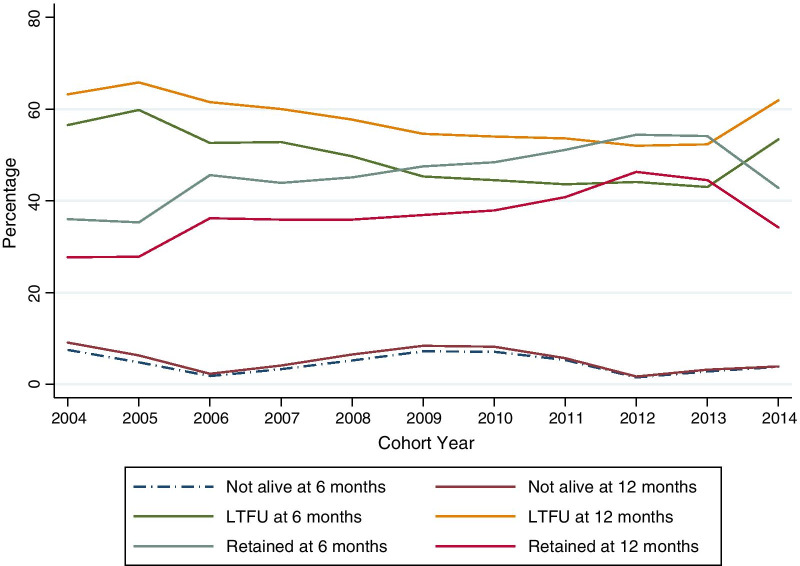
Pre-ART cohort outcomes between 2004 and 2014

The three outcomes assessed were retention, LTFU, and mortality at both 6 and 12 months. At 6 months, retention in the program ranged between 36.0% in 2004 to a high of 54.0% in 2013 before reducing to 42.8% in 2014. LTFU at 6 months remained around 50.0% for most of the cohort years, while mortality at 6 months was 7.5% in 2004 but reduced to 3.8% in 2014, p < 0.001. At 12 months, retention remained at 50.0% across all the cohorts while mortality reached 3.9% in 2014. The trends seen at 6 and 12 months were statistically significant (p-values < 0.001). Table 2 and Fig. 2 presents these outcomes for the two follow-up assessment points.

**Table 2:** Treatment outcomes for pre-ART cohorts in Central Kenya between 2004 and 2014

	Cohort year											P value
	2004	2005	2006	2007	2008	2009	2010	2011	2012	2013	2014	
*6-Month outcome*												< 0.001
Not alive (n = 647)	19 (7.5)	29 (4.8)	18 (1.8)	75 (3.3)	125 (5.2)	143 (7.2)	109 (7.1)	68 (5.3)	15 (1.5)	23 (2.8)	23 (3.8)	
LTFU (n = 6691)	143 (56.5)	359 (59.8)	536 (52.6)	1196 (52.8)	1190 (49.7)	902 (45.3)	681 (44.5)	563 (43.6)	441 (44.1)	353 (43)	327 (53.4)	
Retention (n = 6441)	91 (36.0)	212 (35.3)	465 (45.6)	995 (43.9)	1081 (45.1)	947 (47.5)	741 (48.4)	659 (51.1)	544 (54.4)	444 (54.1)	262 (42.8)	
*12-Month outcome*												< 0.001
Not alive (n = 764)	23 (9.1)	38 (6.3)	23 (2.3)	92 (4.1)	155 (6.5)	168 (8.4)	125 (8.2)	73 (5.7)	17 (1.7)	26 (3.2)	24 (3.9)	
LTFU (n = 7857)	160 (63.2)	395 (65.8)	627 (61.5)	1360 (60.0)	1382 (57.7)	1088 (54.6)	826 (54.0)	691 (53.6)	520 (52.0)	429 (52.3)	379 (61.9)	
Retention (n = 5158)	70 (27.7)	167 (27.8)	369 (36.2)	814 (35.9)	859 (35.9)	736 (36.9)	580 (37.9)	526 (40.8)	463 (46.3)	365 (44.5)	209 (34.2)

## Discussion

Pre-ART enrollment increased from 254 to 2396 patients between 2004 and 2008. This could have been explained by the adoption of broader diagnostic interventions, particularly, the implementation of Provider Initiated Testing and Counselling (PITC) in 2008 to augment the initial client-initiated approaches [15]. This was followed by a decrease in the absolute pre-ART numbers from 2009 to 2013 following scale-up of decentralized treatment programs and further in 2014, due to the change in treatment guidelines lowering the threshold for initiation of ART to CD4 counts of 500 cells/µl [16].

The majority of pre-ART patients on follow-up were women during the 11 year period with a non-significant change in gender trends. More women than men were infected with HIV as reported in the Kenya AIDS Indicator Survey (KAIS) 2012 [3]. This is consistent with the 2016 county profiles data that reported more women than men as having HIV infection [17]. The explanation could be that women are twice as likely to acquire HIV infection compared to men [18]. Women also tend to encounter more social, cultural and economic inequalities compared to men making them more vulnerable to HIV acquisition [18]. Another explanation for the higher number of women is that fewer men enroll into HIV services and even fewer initiate ART, a finding that has been demonstrated in Central Kenya as well [11, 19]. This is probably due to poorer health seeking behavior, perceived low risk of infection, and poor social support systems for men [20]. These may be addressed through implementation of men friendly and culturally acceptable interventions to improve identification, linkage and enrollment into HIV care [21].

There was no statistically significant difference in the proportions of children, adolescents and adults enrolling into HIV care over the years. This is in contrast to the KAIS 2012 report that suggested a change in the peaks in age specific prevalence in men and women from 2003 to 2012 [3]. Our findings could be a result of having a relatively stable HIV disease burden in Central Kenya over the years. However, the proportion of patients aged between 0 and 4 years reduced from 8.7% in 2004 to 1.1% in 2014 due to effective PMTCT interventions in the region.

Our study demonstrated a reduction in the number of patients enrolled with advanced HIV disease. This is similar to trends seen in several studies in sub Saharan Africa (SSA). A study in Rwanda reported marked improvement in the immune status of patients at enrollment into care [22]. Enrollment into HIV care at earlier disease stages across all HIV testing points was also reported in Ethiopia [23]. Similarly, accessing ART at advanced disease peaked at 35.0% in 2005–06 and fell to 27.0% in 2008–2009 in a study conducted in Kenya, Tanzania, and Uganda [24]. Similarly, in Central Kenya, the proportion of those accessing ART at WHO Stage III and IV disease declined from 30.5% in 2004 to 2.9% in 2012 [11]. Another study of 334,557 adults from several countries in sub-Saharan Africa between 2006 and 2011, reported that the proportion of patients initiating ART with advanced HIV disease decreased from 42.0 to 29.0% [25]. In contrast, a study conducted at Mbarara University between 2007 and 2008 reported that more than one third (40.0%) of patients newly diagnosed with HIV infection were categorized as late presenters, classified as WHO clinical stage III or IV [26].

Enrollment in early stage HIV disease can be explained by the scale up of PITC initiatives in the country resulting in earlier identification prior to onset of AIDS defining illnesses [15]. Further, there was increased access to HIV services through decentralization in the period [27, 28]. The UTT guidelines are expected to translate into earlier ART initiation, resulting in improvement in morbidity and mortality [29].

This study reported a reduction in HIV patients on TB treatment over time while those screening positive for TB signs increased over time. This is similar to findings from the 2016 Kenya TB prevalence survey [30]. Similarly, a study conducted among 274 patients who were asymptomatic for TB and ART naïve reported a high prevalence of subclinical TB in HIV-1 infected patients [31].

Pre-ART retention, LTFU and mortality outcomes at 6 and 12 months improved from the year 2013 onwards compared to years 2006–2012 even though mortality slightly increased from 2012 to 2014. The improvement in later cohorts could be due to improved health systems and revision of the Kenya HIV treatment guidelines in line with emerging evidence such as replacement of stavudine (D4T) with tenofovir (TDF) as the preferred first line resulting in better toxicity profile and hence better adherence as well as raising the CD4 cut-off at ART initiation from 350 to 500 cells/mm^3^ [16, 32]. Despite the improvement in later cohorts, retention in this study averaged 40.0–50.0% at both 6 and 12 months of follow-up. This is similar to what has been reported by other studies. A systematic review in SSA on retention of pre-ART patients from time of HIV testing to ART eligibility reported that the median proportion that was retained between HIV testing and CD4 testing was 59.0% and between CD4 testing and ART eligibility was 46.0% [33]. In contrast, some studies have shown lower retention among the pre-ART population. A study conducted in Nigeria among 5320 patients reported 12 month retention of 23.4% among pre-ART patients seen at 37 health facilities [34]. Lower retention in pre-ART could be due to the asymptomatic state of early HIV disease with patients not perceiving themselves as requiring care. This suggests the challenges of retaining relatively well or asymptomatic patients in treatment programs.

LTFU was noted to be a major contributor to attrition averaging at 50.0% for most cohort years at both 6 and 12 months. The high LTFU reported in our study is similar to data reported by other studies conducted in SSA. A prospective cohort study of 530 clients registering for HIV care between July 2008 and August 2009 at Kilifi District Hospital in Kenya and followed up for 6 months recorded a LTFU rate of 33.6% [35]. In Ethiopia, a single centre study among 626 patients enrolled in pre-ART care between 2010 and 2013 reported that the LTFU was 28.4% after a median follow-up of 6 months [36]. A systematic review and meta-analysis conducted in SSA on pre-ART patients followed up from time of HIV diagnosis to initiation of ART regardless of the number of months of follow-up, reported that among patients eligible for ART, 24.6% were LTFU whereas among ineligible patients 54.2% were LTFU [37].

The high rates of LTFU among ineligible patients may suggest the difficulty of retaining asymptomatic patients in care especially due to lack of motivation to attend clinic in absence of ART. A study in Western Kenya reported higher rates of disengagement from care among those on pre-ART who reported feeling well and therefore not requiring care. Among the same group, re-engagement to care was limited by ack of locator information hampering tracing efforts [38]. Further, patients reported as LTFU may have died and therefore could have been wrongly classified. Other reasons for the high LTFU may be self-referral [39], stigma and denial of HIV status, non-disclosure to sources of social support including peers and family [40]. Personal drive has also been described as a likely barrier or facilitator of engagement and retention in care [41]. Health facility barriers to retention have also been reported in a Western Kenya study, including lack of patient incentives, poor provider-patient interactions, access, and health facility related stigma [42].

The other contributor to attrition was mortality. Our study demonstrated a reduction in mortality over time from earlier cohorts to later cohorts. The mortality rate also generally remained low throughout the cohorts years. The low mortality points towards earlier diagnosis of HIV disease as well as earlier treatment. Similar to our study, a study conducted in Nigeria reported that 5.9% of pre-ART patients died during the first 12 months of follow-up [34]. A study in Western Kenya also found proportionaly lower mortality among pre-ART patients who were traced back to care compared to those on ART [38]. In contrast, some studies from SSA have reported higher mortality. A study conducted in Cote d’Ivoire among 860 patients during the pre-ART period, reported an increase in mortality with reduction in CD4 cell counts [43].

The main advantage of this study was the use of a large dataset with patients data collected over a long period of time which allows for better inference. However there were some limitations which included poor documentation for patients who may have self-transferred or died. This may have resulted in an overestimation of LTFU outcome. Another limitation was missing data where no WHO staging and TB status was documented during the study period which could have affected the results. There is also a possibility that the results may have been influenced by the natural maturation of the program and improvements in HIV service delivery over time. Data were routinely collected and entered into the electronic system and could have included transcription errors. Lack of unique patient identifiers limited the capacity to track patients through various service delivery points and even across various healthcare facilities. Lack of a comparative group among those who initiatied treatment also limits inference of the findings in this study.

## Conclusions

Patients were enrolled at earlier WHO clinical stage over time. This suggested improvements in diagnosis during the follow-up period through interventions such as PITC scaled-up in 2008/2009. Retention and mortality outcomes at 6 and 12 months improved over the 11 year follow-up period. However, as the proportion of asymptomatic patients increased, retention decreased in the later cohorts. The general trends showed a decrease in mortality outcomes at 6 and 12 months with a slight increase in later years mirroring the reduction in retention. There was also a notable decrease in the proportions of children aged 0–4 years enrolled during the period suggesting the contribution of PMTCT interventions in the region. Overall, our findings demonstrate the challenges of retaining asymptomatic patients during program scale-up. It is expected that pre-ART patient numbers will reduce with the scale-up of UTT in Kenya since 2016. We recommend that programs continue to monitor outcomes for patients who fail to initiate ART in the UTT era.

## Data Availability

Data used for this analysis is a deidentified dataset of individual level routine HIV care and treatment data and is not currently publically available as it is property of Ministry of Health and Government of Kenya. However, the dataset can be obtained from the corresponding author based on a reasonable request.

## Abbreviations

ART: Antiretroviral therapy
CDC: Centers for Disease Control and Prevention
LTFU: Loss to follow-up
PEPFAR: President’s Emergency Plan for AIDS Relief
PLHIV: People living with HIV
PMTCT: Prevention of mother-to-child transmission of HIV
PITC: Provider initiated testing and counselling
SSA: Sub-Saharan Africa
TDF: Tenofovir
TB: Tuberculosis
WHO: World Health Organization
